# Effects of reboxetine and citalopram on appraisal of infant facial expressions and attentional biases

**DOI:** 10.1177/0269881111421970

**Published:** 2012-05

**Authors:** Alan Stein, Susannah Murphy, Adriane Arteche, Annukka Lehtonen, Allison Harvey, Michelle G Craske, Catherine Harmer

**Affiliations:** 1 Department of Psychiatry, University of Oxford, Oxford, UK; 2 Department of Psychology, University of California, Berkeley, CA, USA; 3 Department of Psychology, University of California, Los Angeles, CA, USA

**Keywords:** Antidepressant, cognitive bias, depression, facial expression

## Abstract

Difficulties in mother–child interaction are commonly observed in the context of postnatal depression. These difficulties may result in part from the negative cognitive bias present in depression, which may in turn lead to biased negative perceptions of the infant: in particular, these biases encompass the negative appraisal of facial expressions. Given the important role of early mother–child interactions in child development it is vital to investigate potential interventions that might be beneficial in ameliorating the negative cognitive bias. This study aimed to examine the effects of two different antidepressants (reboxetine and citalopram) on the appraisal of infant facial expressions of emotion using a faces rating task, and on attention to infant emotion using an attentional probe. Thirty-nine volunteers were randomly assigned to a double-blind 7-day intervention with either placebo, citalopram or reboxetine. There were significant positive effects on the appraisal of facial expressions; participants assigned to the placebo group rated positive faces less positively than those either in the citalopram or in the reboxetine groups. However, there was no evidence that these drugs had an effect on attentional vigilance. If antidepressants are able to help a mother to perceive her infant’s facial expressions as more positive, this may lead to more positive interactions, thereby potentially mitigating the negative effects of depression on infant development. These findings should be treated with caution until replicated in larger and clinical samples.

## Introduction

Maternal postnatal depression is associated with difficulties in mother–child interactions (Field, 1995) and, in particular, with poor maternal responsiveness (Murray et al., 1993; Stanley et al., 2004). Research suggests that these difficulties might be due, in part, to the fact that depressed mothers perceive their infants’ behaviour more negatively than non-depressed mothers (Field et al., 1993). As most early infant communication derives from facial expressions (Papousek and Papousek, 1977), one possible explanation is that mothers with postnatal depression are differentially affected by, and are less sensitive to, infant facial expressions and thus appraise them more negatively.

Such a pattern of negative perceptions is believed to be a function of broader information processing biases that are commonly observed in depressed people (Mathews and MacLeod, 2005; Donaldson et al., 2007). For example, depressed mood has been shown to be associated with negative interpretations of ambiguous facial expressions (Hale, 1998; Lee et al., 2008). Also, depression has been associated with an attentional bias towards negative emotional cues in facial expressions (i.e. sad emotions) (Gotlib et al., 2004), although evidence for attentional biases towards negative stimuli in depression has not been consistent (Bouhuys et al., 1996; Yoon et al., 2009). Interestingly, one study which has examined pregnant women’s responses to infant facial expressions (Pearson et al., 2010) showed that whereas non-depressed pregnant women revealed an engagement bias towards distressed infant faces, those who were depressed tended to actually disengage quicker from the images. The authors suggest that an avoidance mechanism may therefore operate.

Two of our own recent studies focused on processing biases in postnatally depressed mothers. In the first study (Stein et al., 2010), we found evidence of an appraisal bias, as mothers suffering from postnatal depression were more likely to rate sad infant faces as more negative than controls; in particular, if the faces were shown for a longer period of time (2000 ms). In the second study, using a morphed faces task, mothers with depression were less likely to accurately identify happy infant faces than controls, although there were no group differences in the processing of sad faces (Arteche et al., 2011). These findings in relation to the processing of positive faces are consistent with studies using morphed adult faces (e.g. Joorman and Gotlib, 2006), and with other recent evidence that indicates that depression is characterized by difficulties in processing positive affect perhaps even more than by biases in processing of negative affect (Deveney and Deldin, 2004; Surguladze et al., 2004).

Given the crucial role of early mother–child interactions in later child development (Yarrow et al., 1984), it is vital to investigate potential interventions that might help to ameliorate the negative cognitive bias and, in particular, the negative effects of depression on the appraisal of facial expressions. To date, most studies using pharmacotherapy for depression have focused on the effects of antidepressants on negative mood. More recently, research using healthy volunteer models has demonstrated that both selective serotonin reuptake inhibitors (SSRIs) and selective noradrenaline reuptake inhibitors (SNRIs) have direct effects on the processing of emotional information, in particular reducing the processing of negative emotional material, such as fearful and angry faces, and increasing the perception of, and memory for, positively valenced emotional material. For example, seven days treatment with either the serotonergic antidepressant, citalopram, or the noradrenergic antidepressant, reboxetine, has been shown to positively bias the appraisal of ambiguous facial expressions, with decreased recognition of negative facial expressions such as fear and anger (Harmer et al., 2004). In addition, SSRIs, but not SNRIs, have been shown to be useful in the treatment of anxiety, potentially by having an effect on emotional processing biases that are relevant to anxiety (Dhillon et al., 2006). For example, Murphy et al. (2009) have shown that the SSRI citalopram, but not the SNRI reboxetine, reduces attentional vigilance towards threat-related stimuli.

The current study aimed to examine the effects of two different antidepressants (reboxetine and citalopram) on the processing of infant-related emotional information in a group of healthy volunteers. In particular, we sought to explore the effects of these antidepressants on the appraisal of infant facial expressions of emotion using a faces rating task, and on attention to infant emotion using an attentional probe task. It was hypothesized that both drugs would be associated with a positive bias in the appraisal of infant faces of emotion (i.e. that positive faces would be rated more positively) and that citalopram, but not reboxetine, would be associated with a reduction in attentional bias to negative infant emotion.

## Methods

### Participants

Participants were recruited via adverts in university departments. All volunteers were screened and those with a current or previous history of psychiatric disorder (assessed using the Structured Clinical Interview [SCID] for the Diagnostic and Statistical Manual of Mental Disorders, fourth edition [DSM-IV]); history of alcohol or other substance abuse or dependence (assessed using SCID criteria); pregnancy or lactation; history of medical disorder; and current usage of any medication other than oral contraception were excluded from the study. A total of 43 participants (20 male, 23 female) were recruited and randomly assigned to a double-blind 7-day intervention with either (i) placebo, (ii) citalopram (20 mg/day) or reboxetine (4 mg b.i.d.). Seven-day treatment was used because previous research had shown the effects of antidepressant treatment on cognitive biases within this time scale (Harmer et al., 2004). The three groups were matched for gender, age (mean = 24.24, standard deviation [SD] = 3.18) and verbal intelligence quotient (accessed using the National Adult Reading Test; mean = 117.8, SD = 4.79). Four participants were occasional smokers (e.g. less than two cigarettes a day). All participants were students and, as far as we are aware, none had children although this question was not specifically asked. A previous paper has reported on attentional vigilance to threat using this sample (see Murphy et al., 2009).

Thirty-nine participants had complete data on the attentional probe task and 36 completed the face ratings task.^1^ Demographic characteristics of those with complete data on each of the tasks (see Table 1) did not significantly differ from the full study sample. Approval for the study was obtained from the local ethics committee and all volunteers gave written consent.

**Table 1.** Demographic characteristics of participants and mood assessment.

			Citalopram	
	Placebo (*n* = 14)	Reboxetine (*n* = 12)	Dot probe (*n* = 13)	Face ratings (*n* = 10)
*Demographics*				
Sex (*n* male)	7	6	7	5
Age (mean/SD)	23.64 (2.73)	23.83 (1.95)	24.23 (3.03)	23.80 (2.78)
Verbal IQ (mean/SD)	119.31 (4.84)	117.97 (4.44)	116.20 (5.11)	116.41 (4.74)
*Mood assessment pre-treatment (mean/SD)*				
STAXI				
State	31.54 (7.38)	32.45 (7.03)	31.77 (6.93)	31.80 (7.77)
Trait	33.14 (6.51)	29.91 (4.25)	32.33 (6.92)	32.80 (7.49)
Buss–Durkee Hostility	22.38 (5.84)	24.92 (12.13)	24.38 (5.22)	24.10 (5.91)
DAS	115.23 (21.31)	121.67 (29.61)	130.08 (24.73)	131.20 (24.76)
*Mood assessment post-treatment (mean/SD)*				
STAXI				
State	33.46 (10.51)	30.27 (6.29)	32.00 (9.75)	32.00 (11.14)
Trait	34.64 (9.49)	30.73 (5.06)	33.33 (7.77)	33.10 (8.54)
Buss–Durkee Hostility	21.00 (6.67)	24.08 (12.32)	24.53 (6.73)	23.70 (7.39)
DAS	117.69 (24.07)	120.58 (26.48)	130.15 (26.11)	130.30 (27.72)

SD = standard deviation, IQ = intelligence quotient, STAXI = State–Trait Anxiety Inventory, DAS = Dysfunctional Attitudes Scale.

### Assessment of mood, anxiety, hostility and dysfunctional attitudes

These were recorded at baseline and on day 7 of treatment using the Beck Depression Inventory (Beck et al., 1961), the State-Trait Anxiety Inventory (Spielberger et al., 1983), the Buss Durkee Hostility Inventory (Buss and Durkee, 1957), and the Dysfunctional Attitudes Scale (Weisman and Beck, 1978). In addition, on the last day of treatment participants completed the dot probe task and the face ratings task.

### Stimuli and procedures

Two tasks were presented on a 12-inch laptop monitor. The attentional probe task was administered first, followed by the face ratings task. Before taking part in these tasks, all volunteers had already completed a battery of cognitive tasks in this session, but had not been previously exposed to infant faces of emotion.

#### Attentional Probe Task

In this task, pairs of photographs of infant and adult faces were presented. Infant faces were drawn from a database of digital photographs of 27 infants who were filmed at home (see Kringelbach et al., 2008 for details), whilst adult faces were taken from the Ekman database of faces (Ekman and Friesen, 1975; see Kringelbach et al., 2008 for details). Faces were shown as greyscale images and matched for size and luminosity. On half of the trials, pairs of adult faces were displayed and on the other half of the trials, pairs of infant faces were displayed. Each face pair comprised one emotional (sad/happy) face and one neutral expression of the same individual. Thus, there were four types of face pairs: happy/neutral adult (HA), happy/neutral infant (HB), sad/neutral adult (SA), sad/neutral infant (SB). There were 48 trials in total (12 HA; 12 SA; 12 HB; 12 SB). On each trial, one of the faces appeared to the left of the screen and the other to the right of the screen. On each trial, the face pair was presented for 1000 ms and immediately followed by a probe, which appeared in the centre of the location of one of the preceding faces. The probe was one or two small dots. Participants were required to report whether there was one or two dots on the screen by pressing a labelled key on the keyboard. The dots remained on the screen until the participants had made their response. Participants were asked to respond as quickly and as accurately as possible. The task was fully counterbalanced for emotion location, probe location and probe type.

#### Infant Face Ratings Task

Fifty infant faces were presented, ten for each of five emotion conditions (positive, muted positive, neutral, muted negative, negative). Muted faces were chosen to be midway in expression between neutral and positive and muted negative faces were chosen to be midway between neutral and negative. Images were drawn from the same database used in the dot probe task and similarly shown as greyscale images and matched for size and luminosity. Participants were required to rate each facial expression using a Likert-scale ranging from −9 (very negative) to +9 (very positive). Keyboard buttons were labelled accordingly and only designated response keys were registered by the computer. After six practice trials, each face was shown twice in randomized orders, for a short (100 ms) and for a long (2000 ms) duration in order to investigate effects of length of exposure (Frewen and Dozois, 2005; Donaldson et al., 2007). Average scores were computed for each facial expression in each length condition. The hypothesis was that both antidepressants would be associated with a positive bias, meaning that participants would rate positive infant faces more positively.

#### Attentional Probe and Face Ratings Analysis

Data were analysed using treatment group (e.g. placebo vs. reboxetine vs. citalopram) as between-subjects factor and dot probe and face ratings’ variables as within subjects. At the outset, multivariate analyses of variance (MANOVAs) were computed, but because of the relatively small sample size and the resultant lack of power, individual ANOVAs were also computed to investigate group effects in relation to our particular hypotheses.

## Results

### Mood, anxiety, hostility and dysfunctional attitudes

In line with previously reported data (Murphy et al., 2009) there was no evidence of significant change in any of the assessed measures for any of the groups (all *p*-values >.20).

### Attentional probe

Trials with an incorrect indication of the probe were removed (1.8% of all trials). In order to minimize the influence of outliers, trials with response times <200 ms or >2000 ms were also excluded (0.2% of all trials with correct indication of the probe). For each condition, mean attentional vigilance scores were computed as the difference between the average response time in the trials in which the probe was congruent with the position of the emotional faces, and the average response time in the trials in which the probe was congruent with the position of the neutral faces. Therefore, positive scores suggest an attentional vigilance towards the emotional stimuli, whilst negative scores indicate attentional bias away from the emotional stimuli. Zero scores suggest no bias towards the emotional face. Table 2 shows mean and standard deviations for all conditions by group. All variables met normality assumptions.

**Table 2.** Dot probe: means and standard deviation (SD) for bias scores by group.

	Mean (SD) [95% confidence interval]		
	Placebo (*n* = 14)	Citalopram (*n* = 13)	Reboxetine (*n* = 12)
Happy adult bias	−7.29 (29.49)	0.50 (38.53)	2.54 (15.43)
	[−23.39 to 8.80]	[−16.20 to 17.21]	[−14.84 to 19.93]
Happy infant bias	0.08 (24.90)	0.74 (32.54)	3.74 (26.44)
	[−15.16 to 15.32]	[−15.07 to 16.57]	[−12.71 to 20.21]
Sad adult bias	11.55 (19.29)	−4.51 (23.10)	6.20 (31.61)
	[−1.92 to 25.02]	[−18.49 to 9.46]	[−8.34 to 20.75]
Sad infant bias	−6.91 (32.39)	3.60 (23.30)	11.64 (18.40)
	[−20.88 to 7.04]	[−10.88 to 18.09]	[−3.43 to 26.72]

A 2 (adult/infant) × 2 (happy/sad) × 3 (control/citalopram/reboxetine) MANOVA was conducted, followed by inspection of individual ANOVAs to explore differences among the three groups in adult and infant faces in each of the specific emotional conditions. For adult faces, the overall MANOVA was not significant (*F*(4, 72) = 0.84, *p* = .50, η^2^ = .04); nor was there evidence of emotion by Group interaction HA (*F*(2, 36) = 0.41, *p* = .67, η^2^ = .02), SA (*F*(2, 36) = 1.45, *p* = .25, η^2^ = .007). Similarly, for infant faces, the overall MANOVA was not significant (*F*(4, 72) = 0.84, *p* = .51, η^2^ = .04) and inspection of individual ANOVAs revealed no significant Group effects in the specific emotions, HB (*F*(2, 36) = 0.06, *p* = .94, η^2^ = .003), SB (*F*(2, 36) = 1.70, *p* = .20, η^2^ = .09.

### Face ratings

Length of presentation and group effects were examined in each emotional condition. Table 3 shows descriptive statistics for each condition and length by group. All variables met normality assumptions.

**Table 3.** Face ratings: means and standard deviation (SD) for each infant face condition and length by group.

		Mean (SD) [95% confidence interval]		
Length of presentation	Emotional condition	Placebo (*n* = 14)	Citalopram (*n* = 10)	Reboxetine (*n* = 12)
Long presentations	Positive	5.70 (0.90) [5.15 to 6.24]	6.58 (1.16) [5.93 to 7.22]	6.55 (0.98) [5.96 to 7.14]
	Muted positive	4.69 (0.81) [4.14 to 5.24]	5.09 (1.25) [4.44 to 5.74]	5.06 (0.99) [4.47 to 5.66]
	Neutral	0.46 (0.73) [0.09 to 0.82]	0.90 (0.66) [0.47 to 1.33]	0.50 (0.62) [0.10 to 0.90]
	Muted Negative	−3.88 (0.47) [−4.38 to −3.39]	−3.84 (1.27) [−4.43 to −3.25]	−3.70 (0.95) [−4.23 to −3.16]
	Negative	−5.54 (0.87) [−6.07 to −5.01]	−5.72 (1.12) [−6.35 to −5.09]	−5.86 (0.97) [−6.44 to −5.92]
Short presentations	Positive	5.13 (0.99) [4.43 to 5.82]	5.69 (1.82) [4.87 to 6.51]	6.26 (1.05) [5.51 to 7.02]
	Muted positive	4.32 (0.99) [3.76 to 4.88]	4.36 (0.85) [3.69 to 5.02]	4.82 (1.21) [4.21 to 5.42]
	Neutral	0.36 (0.79) [0.05 to 0.77]	0.43 (0.74) [0.05 to 0.92]	0.37 (0.73) [0.07 to 0.82]
	Muted negative	−2.70 (1.05) [−3.34 to −2.07]	−2.53 (1.50) [−3.28 to −1.78]	−2.75 (0.99) [−3.45 to −2.07]
	Negative	−4.19 (1.15) [−4.82 to −5.82]	−4.70 (1.41) [−5.44 to −3.96]	−4.47 (0.89) [−5.15 to −3.80]

### Length of presentation effect

A 2 (stimulus duration) × 3 (treatment group) repeated measures ANOVA was performed for each of the five emotional expressions separately to examine the effect of length of stimulus exposure on ratings of emotional faces. Results revealed that in all groups, longer exposure to stimuli was associated with more extreme ratings of all emotional expressions, with stronger effect sizes being observed for negative faces (positive *F*(1, 33) = 11.41, *p* = .002, η^2^ = .26; muted positive *F*(1, 33) = 8.36, *p* = .007, η^2^ = .20; neutral *F*(1, 33) = 6.32, *p* = .02, η^2^ = .16; muted negative *F*(1, 33) = 44.20, *p* < .0001, η^2^ = .57; negative *F*(1, 33) = 45.55, *p* <.0001, η^2^ = .58). This pattern was consistent across all three groups (all interaction terms *p*-values >.20). Given these duration effects, the following analyses of Group × Facial Expression were conducted separately for short and for long durations.

### Treatment group effects

**Figure 1.**
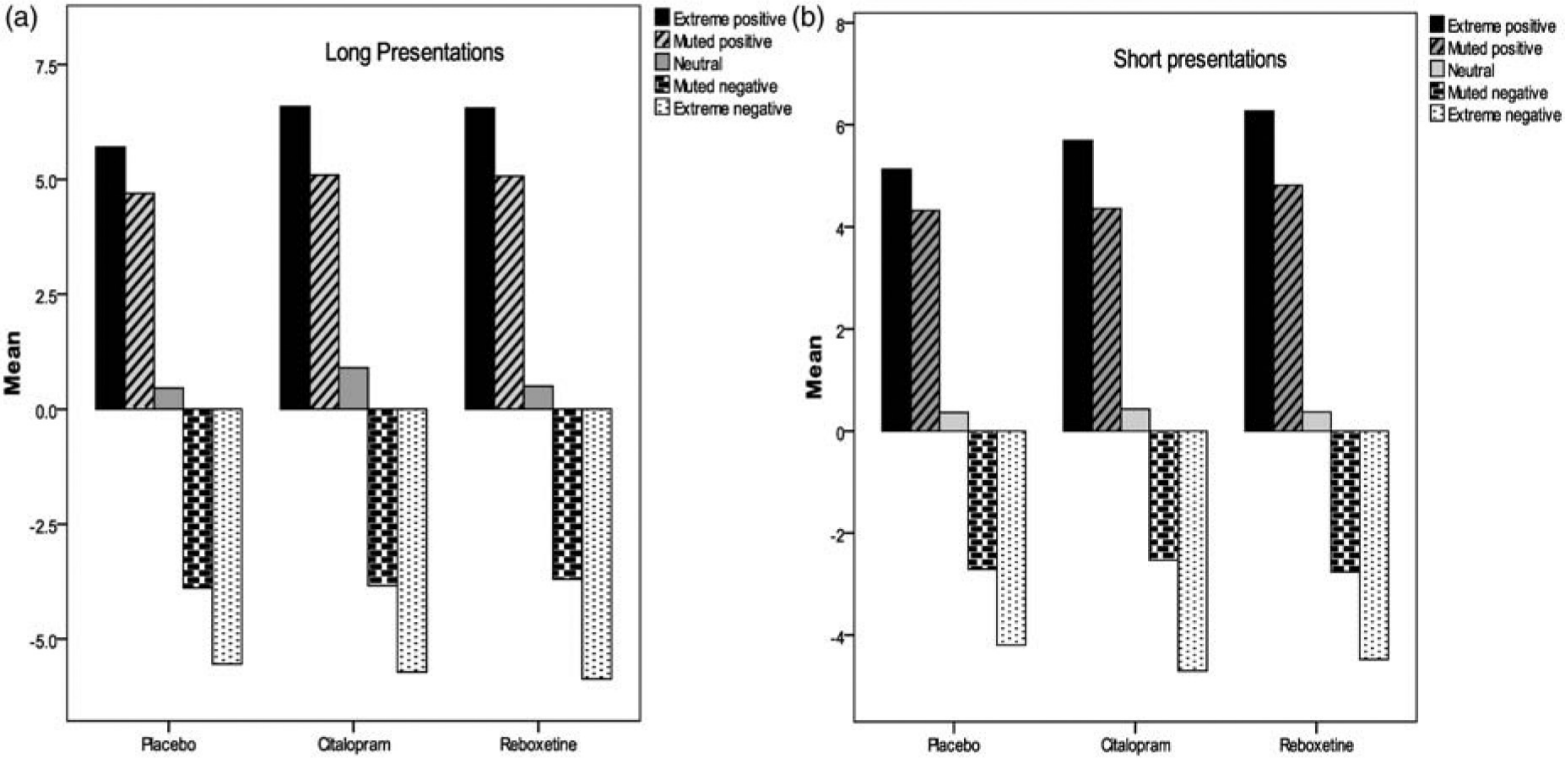
Means by group in the face ratings task for (a) long presentations and (b) short presentations.

In order to investigate specific differences between the placebo group and each of the two drug treatment groups MANOVAs were computed, with Group as the between-group factor, and Emotional Expression as the within subject factor. One MANOVA was conducted on short duration presentations and the other on long duration presentations. Data revealed no overall group effect for either long (*F*(10, 60) = 1.66, NS) or short presentations (*F*(10, 60) = 0.76, NS) on ratings of infant facial expressions of emotion. Given the specific hypothesis that the two antidepressant groups would show a more positive bias in the appraisal of infant expressions (i.e. that they would rate positive faces more positively), we investigated group effects for each emotional expression. There were no group differences in the neutral, negative, muted positive and muted negative faces for either long or short presentations (all *p*-values >.25). However, a significant group effect (*F*(2, 33) = 3.18, *p* = .05, η^2^ = .15) emerged in the positive face-long presentation (see Figure 1a). Pairwise comparisons revealed that participants assigned to the placebo group rated positive faces less positively than those in the citalopram (*p* = .04) and in the reboxetine (*p* = .03) groups. There was no difference between the two treatment groups (*p* = .94). A similar pattern was observed in the positive face-short presentation, although this was not significant (*F*(2, 33) = 2.56, *p* = .09, η^2^ = .13) (see Figure 1b). Pairwise comparisons revealed that participants in the placebo group gave less positive ratings than those in the reboxetine group (*p* = .03). There was no significant difference between placebo and citalopram (*p* = .30) or between the two drug groups (*p* = .30).

## Discussion

This study investigated whether the use of antidepressants in healthy volunteers had an effect on the processing of infant related emotional information (i.e. attention to infant and appraisal of infant facial expressions). In addition, we further examined the possible specificity of the effects of serotonergic (citalopram) and noradrenergic (reboxetine) antidepressants on emotional processing of this kind.

Contrary to our hypothesis, we found no evidence of an SSRI effect on attention vigilance to infant faces of emotion. However, in line with our hypothesis, we did find evidence of effects of both drugs on the appraisal of facial expressions. The absence of significant findings in relation to attentional vigilance is in contrast to the previous report of an effect reduced attentional vigilance towards threat-related stimuli using adult faces following SSRI administration in healthy volunteers (Murphy et al., 2009). One important distinction is that this previous report in healthy volunteers investigated the effect of antidepressants on attentional vigilance to fearful faces, whereas the current study used sad adult and infant faces. It seems plausible to suggest that the lack of effect of the antidepressants in the current study was due to the task not inducing a significant bias in the placebo group and therefore lacking power to detect any drug-related modulation of the bias.

Our findings in relation to appraisal of infants’ faces are interesting in light of previous work which has shown that postnatal depression has a negative effect on the appraisal of infant facial expressions (Stein et al., 2010), and suggests a potential mechanism by which antidepressant drug treatment may mitigate the negative effects of postnatal depression on mother–child interaction. Furthermore, given that these effects were evident for both classes of antidepressants, our findings are consistent with previous work that has shown that both SSRIs and SNRIs have an effect on the interpretation of facial expressions (Harmer et al., 2004). Our current findings are potentially novel given that the effects of antidepressants were observed in relation to infant faces and this has possible clinical implications. Depressed mothers tend to perceive their infants’ behaviour more negatively than non-depressed mothers and this might underlie the difficulties in mother–infant interactions that are commonly observed in depressed mothers (Murray et al., 1993; Stanley et al., 2004). Thus, if, as suggested by our results, the use of antidepressants helps the mother to perceive her infant more positively, this may in turn lead to less dysfunctional and more positive interactions, thereby potentially preventing the negative effects of postnatal depression on infant development. However, this will need to be the subject of further research. It should be noted that while the findings were only significant in relation to the appraisal of positive infant faces, there were also some non-significant changes in relation to negative infant faces (effect size = 0.3) in the expected direction. Our previous study suggests that depression does affect their appraisal of negative faces (Stein et al., 2010) and thus larger studies are needed to test these issues in more detail.

Our study has a number of strengths including a tight experimental design, with healthy volunteers randomized to three different treatment groups including two different antidepressants with different mechanisms of action. Furthermore, infant and adult faces were used, whereas almost all previous studies of attention and appraisal biases in depression have used adult faces only. Nevertheless, the limitations of this study should be considered. First, our sample was relatively small and these findings need to be tested in larger samples, particularly to examine group by face emotional expression interactions. Second, our participants were student volunteers and we did not definitively ascertain that they were not parents; more research is required to investigate whether these findings would hold amongst parents. Third, we chose seven days of antidepressant administration as this has been shown to lead to changes in appraisal of, and attention to, adult faces. Further studies are needed to examine whether a longer period of antidepressant use might lead to a different pattern of changes. Finally, further research needs to confirm these effects in a clinical sample and to investigate whether changes in the appraisal of infant faces has a significant positive impact on mother–infant interactions.

In conclusion, postnatal depression is common, with a prevalence of around 10% (O’Hara, 1997) and a large body of evidence suggests that it is associated with difficulties in mother–child interaction and child development. Recent research indicates that these difficulties arise, at least in part, because of the impact of depression on the mother’s appraisal of her infant’s facial expression. The current study indicates that antidepressants may play a role in mitigating these negative effects by altering the appraisal of infant facial expressions. If these findings hold in larger and postnatally depressed samples, they have important implications for clinical practice.

## Footnotes

1 The three participants who completed the dot probe, but not the face ratings were recruited into the Citalopram group.
